# COVID-19 vaccine hesitancy and influential factors among Thai parents and guardians to vaccinate their children

**DOI:** 10.1016/j.jvacx.2022.100182

**Published:** 2022-06-13

**Authors:** Amornphat Kitro, Wachiranun Sirikul, Ekkamon Dilokkhamaruk, Gowgeat Sumitmoh, Sarisa Pasirayut, Amnart Wongcharoen, Jinjuta Panumasvivat, Krongporn Ongprasert, Ratana Sapbamrer

**Affiliations:** a Department of Community Medicine, Faculty of Medicine, Chiang Mai University, Chiang Mai Province 50200, Thailand; b Faculty of Medicine, Chiang Mai University, Chiang Mai Province 50200, Thailand; c Health Promotion Unit, Faculty of Medicine, Chiang Mai University, Chiang Mai Province 50200, Thailand

**Keywords:** COVID-19, Vaccine-hesitancy parents, Children, Thailand

## Abstract

**Background:**

The ongoing COVID-19 has had a huge worldwide influence. Vaccinations are a crucial component of illness prevention. Thailand's vaccination program for children above the age of five is in its initial stages. Many Thai parents are still unwilling to vaccinate their children with the COVID-19 vaccine. The purpose of this study was to analyze the rate of parental hesitancy and influential variables among Thai parents and guardians living in Thailand.

**Methods:**

This was a cross-sectional survey in Thailand during October and November of 2021. Through online social media networks, an online survey (REDcap) was distributed. Thai parents and guardians with at least one child under the age of 18 were invited to participate.

**Results:**

A total of 1064 responses for analysis. One thousand and sixty-four were available for analysis with 74.4% of those who participated being female. The results found that the parental COVID-19 vaccination hesitancy rate was higher among parents with children under the age of 12 than among parents with children over the age of 12. (56.9% vs 17.1%, p-value < 0.001). Parents were more concerned about vaccination side effects (82.5%), safety (60.0%), and vaccine efficacy (37.2%). Low vaccine reluctance among parent with children age less than 12 years old was associated with higher education (aOR = 0.35, 95 %CI = 0.13–0.93, *p*-value = 0.036) and a favorable attitude regarding covid-19 immunizations (aOR 0.16, 95% CI 0.04–0.67, p-value 0.012)

**Conclusion:**

The majority of Thai parents with children under the age of 12 were hesitant to vaccinate against COVID-19. To minimize Thai parent vaccination hesitancy and increase vaccine uptake among children, the government and health care providers should target this population with evidence-based information, promote positive attitudes toward COVID-19 vaccinations, and encourage educated parents to vaccinate their children at first.

## Highlights

• Parents with children under the age of 12 showed more vaccine hesitancy.
• Parents were most concerned about vaccination adverse effects and safety.
• Low vaccination hesitancy linked with higher socioeconomic level.
• Low vaccination hesitancy linked with positive attitude toward COVID-19 immunization.
• Government and public health authority made parent less hesitant in making decision.

## Introduction

The ongoing COVID-19 pandemic has caused significant impact, with approximately 525 million cases documented globally [1]. There are approximately 4.41 million individuals in Thailand with confirmed COVID-19 infection, with 29,746 deaths resulting from the outbreak including patients returning from abroad [2]. The number of infected cases from newborn to 18-year-old children from the Delta variant of COVID disease in Thailand (April to December 2021) was reported as accounting for 15.6% of all infected cases. During the current Omicron outbreak (January to February), 16.1% of all affected were children. Furthermore, the proportion of children aged 5 to 11 years old gradually increased (6.2 to 6.6%) within a month [3]. Vaccinations are the most reliable method of primary prevention, promoting immunity and inhibiting virus transmission; consequently, Thailand's vaccination program for children 12 to 18 years of age began immunizations against COVID-19 with a Pfizer vaccine in August 2021 and children aged 5 to 12 years old in February 2022 [4]. In May 2022, approximately 78.3%, 76.6%, and 8.4% of 12 to 17-year-old children had their first, second, and third Pfizer vaccination shots, respectively, while 55.0% and 19.3% of children aged 5 to 11 had their first and second Pfizer vaccination doses, respectively [5]. According to the Ministry of Public Health's Epidemiological Surveillance Report, the rate of myocarditis and pericarditis following Pfizer vaccination was 0.09 per 100,000 vaccinees [6].

Following FDA approval in the United States, Pfizer-BioNTech vaccination comprises two doses (30 µg each) separated by three weeks in children under the age of twelve [7]. In phase 2/3 investigations evaluating the efficacy and side effects of the Pfizer-BioNTech vaccine in teenagers aged 12 to 15 years old, it was discovered that the vaccination is nearly 100% effective in preventing COVID-19 infection (95 %CI = 75.3–100%) [8]. Furthermore, the COVID-19 immunization schedule of two Pfizer 10 µg doses given 21 days apart was determined to be 90.7% efficacious in preventing COVID-19 in children aged 5 to 11 (95% CI = 67.7 to 98.3) with no major adverse consequences being detected in the ongoing study [9].

Concerning adverse events, 90.9% were discovered to have local side effects such as discomfort at the injection site, and 90.7% experienced systemic side effects such as fever, tiredness, and headache that resolved in one to two days [7], [10]. Side effects of the Pfizer-BioNTech vaccine, such as myocarditis and pericarditis, were rare. Any serious side effects typically occur on days two to seven following the second dose of this vaccination and are most common in males aged 12 to 17 years old, with 56 to 69 cases per 1,000,000 doses. Females, on the other hand, have a lower incidence, with only four to five instances per 1,000,000 doses. In a short-term trial, a patient who developed myocarditis and pericarditis after receiving the vaccination was treated and recovered; however, long-term studies are still unavailable [11]. Studies in the United Kingdom and Canada found that the extended regimen, which increased the interval between Pfizer-BioNTech doses to 8 to 12 weeks, reduced side effects and increased antibody levels following the second dose in comparison to the short regimen [12], [13]. Only the Pfizer-BioNTech vaccine is approved for use in children aged 5 to 18 years old in the United States [14]. The Royal College of Pediatricians of Thailand recommends this vaccine for children aged 12 to 18 years old, as well as immunization for children with chronic conditions and healthy children using Pfizer-BioNTech 30 µg with two doses administered eight weeks apart. Furthermore, it is recommended that children aged 5 to 12 years old are immunized with Pfizer-BioNTech 10 µg vaccine in two doses spaced eight weeks apart [4], [15].

Many studies were carried out into the vaccine hesitancy rate among parents to vaccinate their children with the COVID-19 vaccine and outcomes demonstrated a range of 13.3–71.7% [16], [17], [18], [19], [20], [21], [22], [23], [24], [25], [26]. 13.3–40.7% of Chinese parents did not want their children to get vaccinated [20], [22], [23] figures similar to American parents (27.3–38.1%) and European parents (39.5–49.0%) [17], [18], [19], [21], [24] Parents' willingness was being influenced by the fact that they recognized their children as high-risk (OR = 2.5, 95% CI = 1.2–5.0), regularly read COVID-19 vaccine-related information (OR = 9.1, 95% CI = 3.2–28.7), believed in the safety of the COVID-19 vaccine (OR = 3.1, 95% CI = 1.3–7.2), and believed the COVID-19 vaccine could indeed prevent COVID-19 (OR = 13.8, 95% CI = 2.5–75.1) [20]. According to a study conducted in Chicago, Illinois, USA, parents who obtained information about COVID-19 vaccine from medical practitioners, the internet, or family members were more likely to vaccinate their children [18]. Furthermore, parents who had already had their children vaccinated or planned to vaccinate their children with the flu vaccine expressed less hesitation [19], [24]. Educated parents who believed in the safety of vaccines improved vaccine acceptance by 1.2-fold (95% CI = 1.1–1.4) [21]. Parents were hesitant to vaccinate their children because they were concerned about side effects, short- and long-term efficacy, safety, or believed that their children did not need to be vaccinated [17], [19], [24], [25]. Furthermore, parents with a low level of education, income, and health literacy are more likely to be vaccine-hesitant [27].

Thailand is now vaccinating children above the age of five with Pfizer-Biotech. The purpose of this study was to evaluate the vaccination hesitancy rate and factors influencing Thai parents and guardians in vaccinating their children with the COVID-19 vaccine. The results will help parents and guardians overcome vaccine hesitation, improve vaccine attitudes, and establish communication strategies in the appropriate population to increase the number of vaccinations sufficient to achieve herd immunity before returning to school, and to stop the spread of COVID-19 as soon as possible to help us all return to normal lifestyles.

## Methods

### Setting and study design

This was a cross-sectional study conducted in Thailand during October and November 2021. An online survey was distributed using online social media platforms. Study data were collected and managed using REDCap electronic data capture tools hosted at Chiang Mai University. REDCap (Research Electronic Data Capture) is a secure, web-based software platform designed to support data capture for research studies, providing: (1) an intuitive interface for validated data capture; (2) audit trails for tracking data manipulation and export procedures; (3) automated export procedures for seamless data downloads to common statistical packages, and 4) procedures for data integration and interoperability with external sources [28], [29]. Bangkok (Thailand's capital city) and Chiang Mai (the second largest province in Thailand) were the primary distribution hubs. The questionnaire was divided into four sections: parent demographic data, child demographic data, parent attitudes regarding COVID-19 vaccination hesitancy, and potential factors influencing COVID-19 vaccine hesitancy. For the face validity of the investigation, the questionnaire was developed from similar studies. It was validated in a 30-person pilot study and was proven reliable and valid by experts. Adult (≥18-year-old) Thai parents/guardians with at least one child under the age of 18 were eligible. The sample size was estimated using data from relevant studies and the sample size calculation formula from N4studies. In the computation, the following parameters were used: an estimated proportion of parent vaccine hesitancy in a systematic review and *meta*-analysis (P) of 0.258 (30), Error (d) of 0.05, Alpha error of 0.01, and Z (0.975) of 1.959964. The calculated N was 509 individuals. A 10% dropout rate was added, resulting in the need to include a total of 560 individuals.

### Questionnaire

The demographic data of parents/guardians and child/children was collected to assess the study group characteristics. Attitudes toward COVID-19 vaccine hesitancy were collected, including vaccine efficacy, how the vaccine is important for their child's health, their child in the community, reliability of COVID-19 vaccine information from healthcare professionals, following COVID-19 vaccination instructions, and concerns regarding side effects. Data pertinent to the most influential people for vaccine hesitancy, vaccine concerns, vaccine manufacturer preference, need for confirmation of vaccine safety, vaccine efficacy threshold for acceptance, and acceptable rate of mild and serious vaccine side effects was collected for potential factors of COVID-19 vaccine hesitancy. In this study, the hesitancy of COVID-19 vaccination was defined as the unwillingness or whether they were unsure to have their child vaccinated in terms of the proportion of the study population. Attitude towards COVID-19 vaccination hesitancy was measured using the Likert scale which was classified into five levels from the highest degree (5) to the lowest degree (1). Agree and strongly agree were categorized as having a favorable attitude, whereas neither agree nor disagree, disagree, and strongly disagree were categorized as having a negative attitude. The reliability test of six questions for assessing the attitude of parent towards COVID-19 vaccination hesitancy had a Cronbach’s coefficient alpha of 0.8423.

### Statistical analysis

All statistical analyses were conducted using the STATA statistical software program (Stata Corp. 2019, Stata Statistical Software: Release 16, Stata Corp LLC, College Station, TX, USA). Descriptive statistics including percentage, mean, and standard deviation (SD.) were used to evaluate quantitative data from the questionnaire. Independent student T-test and chi-square test were used for analyzing the differences in demographic data between children under 12 years old and above 12 years old. Univariate and binary logistic regression were analyzed for determining the factors associated with COVID-19 vaccine hesitancy. Adjusted odds ratios (aOR) and 95% confidence intervals (95 %CI) were presented. The statistical significance was set at 0.05.

**Fig. 1:**
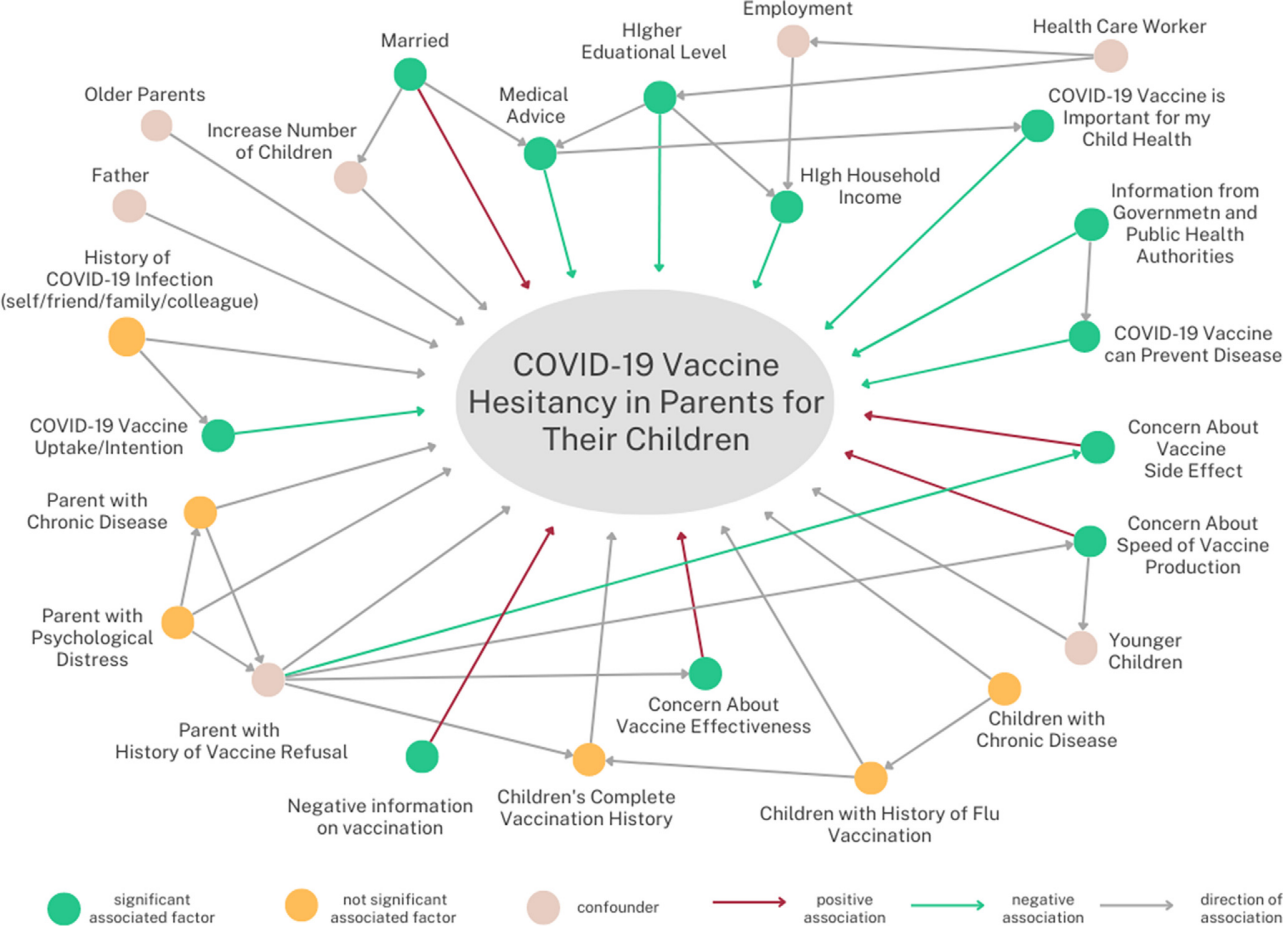
Factors associated with COVID-19 vaccine hesitancy in parents for their children.

The variables which were considered to include to the model of binary logistic regression were shown in Fig. 1.

### Ethics consideration

This study was approved by the Human Ethical Committee at the Faculty of Medicine, Chiang Mai University (no.442/2564), 15 October 2021.

## Results

This survey received 1,240 responses from Thai parents and guardians. A total of 1064 responses were available for analysis. The mean age of the parents was 44.6 (SD. = 8.2) years old. 74.4% (n = 792) of the participants were female. Single parent households accounted for 69.9% of all households (n = 744), with an average of four individuals living together. Around 51% of parents had a single child, with the minority having 2–4 children (47.6%). 73.0% of subjects were married. 59.6% of all parents/guardians had a college degree and above with 77.3% being full-time employees, and 14.5% of all participants were health care workers. 52.9% of all the parents had a household monthly income of 10,001–60,000 THB (300–1,800 USD). There were 71.8% of parents whose friends and colleagues had a COVID-19 positive history and 8.4% who had had a family member die as a result of COVID-19. 85.2% of parents had no history of vaccination refusal (Table 1).

**Table 1:** Parent demographics data (n = 1064).

**Characteristics**	**Mean ± SD. or n (%)**
Parent age (year), mean ± SD	44.6 ± 8.2
Parent gender	
Female	792 (74.4)
Male	272 (25.6)
Single parent household	744 (69.9)
No. of people living in the same house, mean ± SD	4 ± 2
No. of children in the family	
1	535 (50.3)
2–4	500 (47.0)
>5	29 (2.7)
Parent marital status	
Single	169 (15.9)
Married	777 (73.0)
Divorce	83 (7.8)
Widowed	35 (3.3)
Education status	
Primary school	210 (19.7)
High-school or vocational certificate	220 (20.7)
College degree, university	451 (42.4)
Master or above	183 (17.2)
Household income (Thai Baht)	
<10,000	181 (17.0)
10,001–60,000	563 (52.9)
60,001–100,000	185 (17.4)
More than 100,000	135 (12.7)
Employment status	
Employed	823 (77.3)
Unemployed	241 (22.7)
Health care workers	154 (14.5)
Chronic disease	273 (25.7)
History of psychological diagnosis	14 (1.3)
History of COVID-19 positive	
Yourself	42 (4.3)
Family member	121 (12.3)
Friend	321 (31.8)
Colleague	398 (40.0)
History of death in the family due to COVID-19, family member loss due to COVID-19	89 (8.4)
Any vaccine refusal history	
Yes	157 (14.8)
No	907 (85.2)

SD., standard deviation.

Four hundred and ninety-one children were under 12 years old, the average age of the people in this group being 7.1 ± 3.0 years old, 47.7% of them were female. Most of the children (70.6%) were going to school or daycare and 7.3% of children had long-term illnesses. There were 1,271 children over the age of twelve. In this group the mean age was 16.3 ± 4.3 years old, 47.4% of them were female, the majority, 69.9% were going to school or daycare, and 9.6% had chronic illnesses.

The majority of children had had their routine vaccines (94.7% and 85.6% in above 12 years old and less than 12 years old, respectively). Children over 12 years old (34.6%) and 59.8% of children under 12 years old had had the flu vaccine in the last season (Table 2).

**Table 2:** Children demographics data

**Characteristics**	**< 12 years (n = 493)**		**≥ 12 years (n = 1,271)**		***p-*value**
	**n**	**%**	**n**	**%**	
Gender					
Female	235	47.7	602	47.4	0.915
Male	258	52.3	669	52.6	
Attending in person school or day care	348	70.6	888	69.9	0.817
Had chronic diseases	36	7.3	122	9.6	0.138
Complete all their routine vaccines	422	85.6	1,203	94.7	<0.001**
Received flu vaccine last 12 months	295	59.8	440	34.6	<0.001**

** *p*-value < 0.01.

**Fig. 2:**
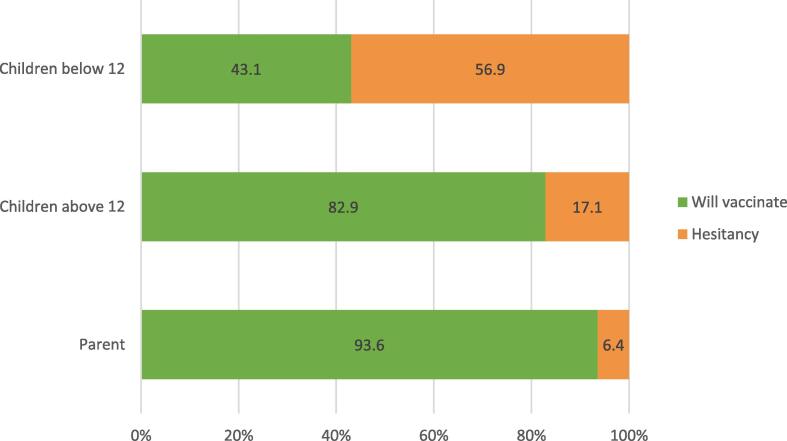
Percentage of parents and guardians willing to vaccinate selves and their children against COVID-19.

56.9% of parents/guardians who had a child younger than twelve were hesitant that they would want to allow their child to be vaccinated with a government-provided vaccine, while 17.1% of parents of children over 12 years old were not confident that they would let their child have the COVID-19 vaccine. Only 6.4% were certain they were hesitant to have the vaccine themselves if it was available (Fig. 2).

**Fig. 3:**
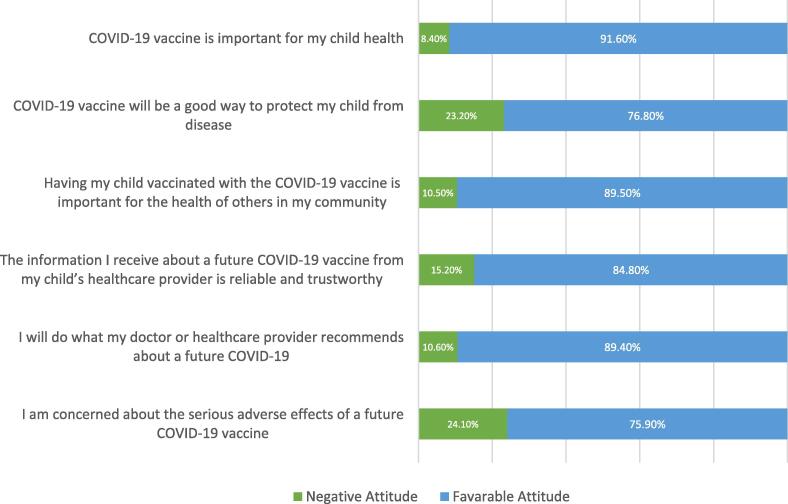
COVID-19 vaccine hesitancy attitudes among Thai parents and guardians.

As shown in Fig. 3, 91.6% of parents and guardians agreed that the COVID-19 vaccine was important for their children. 76.8% of Thai parents agreed with the protection potency of the COVID-19 vaccine, 89.5% agreed that the COVID-19 vaccine was important for the health of others in the community, 84.8% believed that information about COVID-19 vaccine in the future was reliable and trustworthy, and 89.4% had confidence in the vaccine when doctors recommended, and 75.9% were concerned about serious adverse effects of future COVID-19 vaccine.

Table 3 shows the parental concerns about the COVID 19 vaccination. Approximately 82.5% were concerned about vaccine side effects, 60% were concerned about vaccine safety, 37.2% were concerned about the lack of long-term study results on vaccine efficacy. People who influenced parents' decisions were mostly health care providers (81.7%), pediatricians (36.2%), and friends or family (31.4%), with 81.6% of information coming from health care providers, 66.7% from the internet/social media/podcast, 38.3% from family/friend/word of mouth, and 37.8% from TV/newspaper/radio. The most chosen vaccination type was the mRNA vaccine, with Pfizer accounting for 71.8% and Moderna accounting for 13.7%, with acceptable risk of adverse effects (myocarditis/pericarditis), specifically 1 in a million, 1 in 100,000, and 1 in 10,000 were 50.1%, 36.0%, and 13.9%, respectively. 61.5% of parents and guardians would postpone their child/COVID-19 children's vaccination for one to three months.

**Table 3:** Concern and influential factors for COVID-19 vaccine acceptance in parents/guardians

**Influential factors**	**Total = 1064**	
	***n***	**(%)**
**Concern among responders**		
Anxiety about vaccine side effects	878	82.5
Concern about vaccine safety	638	60.0
Lack of long-term study results about vaccine efficacy	396	37.2
Not sufficient evidence about vaccine	324	30.4
Child individual risk factors/previous vaccine reactions	206	19.4
Not being widely used yet/speed of vaccine production/need more information or observation	193	18.1
Not believing in the effectiveness of vaccines	123	11.6
Not being afraid or anxious about COVID infection/ low or no risk of infection	107	10.1
Concern about excipient in the vaccine	103	9.7
Distrust companies developing vaccines	47	4.4
**Source of influences to make decision**		
Health care providers	869	81.7
Paediatrician	385	36.2
Friends or family	334	31.4
Information from government and public health	314	29.5
School requirement/ travel sport or activity participation requirements	284	26.7
**Source of information**		
Health care providers	868	81.6
Internet/social media/podcast	710	66.7
Family/friend/word of mouth	407	38.3
TV/newspaper/radio	402	37.8
Government agency	343	32.2
**Vaccine manufacturer preference for child/children**		
Pfizer	764	71.8
Moderna	146	13.7
Sinopharm	71	6.7
Novavax	35	3.3
AstraZeneca	25	2.3
Sinovac	5	0.5
**Accepted risk of serious side effects (myocarditis/pericarditis)**		
1:10,000	148	13.9
1:100,000	383	36.0
1:1,000,000	533	50.1
**Prefer to delay vaccination**	494	46.6
At least waiting duration		
1 month	137	27.7
3 months	167	33.8
6 months	93	18.8
1 year	97	19.6

Table 4 shows the parental factors associated with COVID-19 vaccine hesitancy for children under the age of 12 years. Parents with a monthly income between 60,001 and 100,000 THB (aOR = 0.15, 95 %CI = 0.03–0.69, *p*-value = 0.015) and more than 100,000 THB (aOR = 0.16, 95 %CI = 0.03–0.82, *p*-value = 0.027) were less likely to be hesitant about having their children vaccinated. Parents who believed that the COVID-19 vaccine was vital for their child's health (aOR = 0.16, 95 %CI = 0.04–0.67, *p*-value = 0.012) were less likely to be skeptical. Concern about the serious adverse effects of a future COVID-19 vaccine (aOR = 2.71, 95 %CI = 1.49–4.92, *p*-value = 0.001), as well as vaccines that had not yet been widely used, vaccine production speed, and vaccine information that needed more consideration were associated with being more hesitant (aOR = 3.56, 95 %CI = 1.69–7.48, *p*-value = 0.001). Government and public health information was discovered to be the source of information related to making parents less hesitant when making decisions for their children (aOR = 0.53, 95 %CI = 0.29–0.96, *p*-value = 0.035).

**Table 4:** Parental factors associated with COVID-19 vaccine hesitancy for children under 12 years of age by multivariable logistic regression

**Factors**	**aOR**	**95 %CI**	***p*-value**
**Parental characteristics**			
**Age**			
<30 years	(ref.)		
30–45 years	1.45	0.49–4.30	0.506
>45 years	1.21	0.39–3.74	0.741
**Male**	0.88	0.43–1.79	0.725
**Single parent household**	0.73	0.44–1.23	0.241
**Parent marital status**			
Single	(ref.)		
Married	2.70	1.22–5.96	0.014*
Divorce	1.82	0.52–6.36	0.347
Widowed	1.40	0.12–15.61	0.787
**Education status**			
Primary school	(ref.)		
High-school or vocational certificate	0.39	0.12–1.29	0.122
College degree, university	0.35	0.13–0.93	0.036*
Master or above	0.48	0.17–1.39	0.176
**Household income (Thai Baht)**			
<10,000	(ref.)		
10,001–60,000	0.25	0.06–1.12	0.070
60,001–100,000	0.15	0.03–0.69	0.015*
>100,000	0.16	0.03–0.82	0.027*
**Healthcare personals**	1.55	0.77–3.08	0.217
**COVID-19 experience**			
**History of COVID-19 positive**			
Yourself	0.91	0.22–3.73	0.890
Friend	0.93	0.52–1.66	0.794
Family member	1.07	0.42–2.67	0.892
Colleague	0.60	0.33–1.11	0.105
**History of death in the family due to COVID-19, family member loss due to COVID-19**	0.86	0.28–2.65	0.795
**Willing to Receive COVID-19 vaccine**	0.50	0.07–3.58	0.491
**Attitude to COVID-19 vaccine (agree to strongly agree)**			
COVID-19 vaccine is important for my child health	0.16	0.04–0.67	0.012*
COVID-19 vaccine will be a good way to protect my child from disease	1.65	0.85–3.20	0.138
Having my child vaccinated with the COVID-19 vaccine is important for the health of others in my community	0.45	0.15–1.31	0.141
The information I receive about a future COVID-19 vaccine from my child’s healthcare provider is reliable and trustworthy	0.67	0.30–1.51	0.331
I will do what my doctor or healthcare provider recommends about a future COVID-19	1.24	0.40–3.82	0.713
I am concerned about the serious adverse effects of a future COVID-19 vaccine	2.71	1.49–4.92	0.001**
**Concern among responders**			
Anxiety about vaccine side effects	1.58	0.79–3.18	0.194
Concern about vaccine safety	1.23	0.70–2.16	0.475
Lack of knowledge about vaccine efficacy	1.34	0.73–2.45	0.343
Not being widely used yet/speed of vaccine production/need more information or observation	3.56	1.69–7.48	0.001**
Child individual risk factors/previous vaccine reactions	1.77	0.90–3.48	0.096
Not being afraid or anxious about COVID-19 infection/ low or no risk of infection	2.22	0.85–5.84	0.105
Not believing in the effectiveness of vaccines	1.15	0.44–3.05	0.775
Lack of long-term study results about vaccine efficacy	1.25	0.70–2.25	0.445
Not sufficient evidence about vaccine	1.02	0.57–1.81	0.953
Distrust companies developing vaccines	3.32	0.78–14.10	0.103
Concern about excipient in the vaccine	0.61	0.24–1.56	0.298
**Source of influences to make decision**			
Pediatrician	0.91	0.53–1.58	0.739
Other health providers	1.68	0.88–3.22	0.116
Seeing/hearing/ experience of friend or family	0.69	0.39–1.23	0.207
Information from government and public health	0.53	0.29–0.96	0.035*
School requirement/ travel sport or activity participation requirements	0.73	0.41–1.28	0.271
Other sources	1.80	0.64–5.04	0.266

aOR, adjusted odds ratio; 95 %CI, 95% confidence interval; ref., reference; * *p*-value < 0.05; ** *p*-value < 0.01

Table 5 summarizes the parental characteristics linked with COVID-19 vaccine hesitancy in children aged 12 and above. Parents who were willing to receive COVID-19 (aOR = 0.25, 95% CI 0.13–0.48, p = 0.001) and significantly believed that the vaccine would be a good way to protect their child from disease (aOR = 0.50, 95 %CI = 0.31–0.81, *p*-value = 0.005) were less likely to be hesitant for their children to receive COVID-19 vaccination. Parents who were more concerned about the serious side effects of the COVID-19 vaccine (aOR = 2.35, 95 %CI = 1.33–4.17, *p*-value = 0.003) and considerably anxious about vaccine side effects (aOR = 0.53, 95 %CI = 0.32–0.86, *p*-value = 0.011) were more likely to be hesitant.

**Table 5:** Parental factors associated with COVID-19 vaccine hesitancy in children 12 years and older by multivariable logistic regression

**Factors**	**aOR**	**95% CI**	***p*-value**
**Parental characteristics**			
**Age**			
<30 years	(ref.)		
30–45 years	0.46	0.18–1.15	0.097
≥45 years	0.45	0.18–1.13	0.090
**Male**	1.61	1.04–2.51	0.034*
**Single parent household**	1.07	0.69–1.66	0.766
**Parent marital status**			
Single	(ref.)		
Married	0.81	0.45–1.46	0.486
Divorce	1.40	0.62–3.16	0.417
Widowed	0.67	0.21–2.11	0.494
**Education status**			
Primary school	(ref.)		
High-school or vocational certificate	0.78	0.42–1.45	0.430
College degree, university	1.02	0.54–1.94	0.946
Master or above	1.21	0.52–2.84	0.659
**Household income (Thai Baht)**			
<10,000	(ref.)		
10,001–60,000	1.02	0.57–1.81	0.955
60,001–100,000	0.94	0.41–2.17	0.893
More than 100,000	0.81	0.31–2.11	0.666
**Healthcare personals**	0.83	0.44–1.59	0.578
**COVID-19 experience**			
**History of COVID-19 positive**			
Yourself	1.31	0.43–4.04	0.633
Friend	0.69	0.43–1.11	0.127
Family member	0.81	0.41–1.60	0.541
Colleague	0.70	0.45–1.09	0.117
**History of death in the family due to COVID-19, family member loss due to COVID-19**	0.84	0.39–1.83	0.668
**Willing to Receive COVID-19 vaccine**	0.25	0.13–0.48	<0.001**
**Attitude to COVID-19 vaccine (agree to strongly agree)**			
COVID-19 vaccine is important for my child health	0.68	0.33–1.40	0.298
COVID-19 vaccine will be a good way to protect my child from disease	0.50	0.31–0.81	0.005**
Having my child vaccinated with the COVID-19 vaccine is important for the health of others in my community	0.75	0.37–1.49	0.405
The information I receive about a future COVID-19 vaccine from my child’s healthcare provider is reliable and trustworthy	0.75	0.41–1.38	0.357
I will do what my doctor or healthcare provider recommends about a future COVID-19	0.66	0.34–1.28	0.218
I am concerned about the serious adverse effects of a future COVID-19 vaccine	2.35	1.33–4.17	0.003**
**Concern among responders**			
Anxiety about vaccine side effects	0.53	0.32–0.86	0.011*
Concern about vaccine safety	1.09	0.70–1.70	0.691
Lack of knowledge about vaccine efficacy	0.86	0.55–1.33	0.488
Not being widely use yet/speed of vaccine production/need more information or observation	1.66	0.97–2.82	0.064
Child individual risk factors/previous vaccine reactions	1.41	0.88–2.25	0.149
Not being afraid or anxious about COVID-19 infection/ low or no risk of infection	1.56	0.85–2.83	0.148
Not believing in the effectiveness of vaccines	1.48	0.82–2.66	0.192
Lack of long-term study results about vaccine efficacy	1.63	0.99–2.68	0.055
Not sufficient evidence about vaccine	0.88	0.51–1.51	0.639
Distrust companies developing vaccines	1.47	0.58–3.72	0.415
Concern about excipient in the vaccine	0.60	0.27–1.32	0.203
**Source of influences to make decision**			
Paediatrician	1.02	0.66–1.58	0.933
Other health providers	1.15	0.66–1.98	0.622
Seeing/hearing/ experience of friend or family	0.84	0.55–1.30	0.443
Information from government and public health	1.03	0.67–1.58	0.886
School requirement/travel sport or activity participation requirements	1.04	0.65–1.67	0.860
Other sources	0.55	0.20–1.55	0.261

aOR, adjusted odds ratio; 95 %CI, ref., reference; 95% confidence interval; * *p*-value < 0.05; ** *p*-value < 0.01.

## Discussion

To the best of our knowledge, this was the first study to assess parental and guardian hesitancy with regard to immunizing their children with the COVID-19 vaccine in Thailand. Approximately two-thirds of parents would hesitate to vaccinate their children under the age of 12, whereas 17.1% would hesitate to vaccinate their child/children ages 12 and above with the current COVID-19 vaccine. Parents' and guardians’ top three concerns were vaccination side effects (82.5%), safety (60.0%), and vaccine efficacy. (37.2%). High socioeconomic status and a positive attitude toward the COVID-19 vaccination were related to less vaccine hesitancy.

Currently, the Thai government is carrying out a COVID-19 mass vaccination campaign for children aged 5 to 18 years old with Pfizer-BioNTech on a two-dose schedule to generate herd immunity among young children and let them return to school. While students under the age of five waited for vaccine approval [4], [15]. Because of the rapid pace of vaccine production and the insufficiency of long-term studies on efficacy and safety in Asian young children, many parents and guardians were unsure whether their children should receive or defer the current COVID-19 vaccine, which the government was providing free to all children aged 5 to 18 years old. Understanding the factors associated with decreased vaccination hesitation in each age group (over 12 years old and under 12 years old) provides crucial insight that would help increase Thai parents' and guardians' willingness to give their children the COVID-19 vaccine.

Our study found that parents with children under the age of 12 were more hesitant to give their children the COVID-19 vaccine than parents with children over the age of 12. (56.9% VS 17.1%). In terms of COVID-19 vaccine hesitancy among parents of different nationalities, Thai parents exhibited similar vaccine hesitancy to parents in the United States (33.0–38.1%), Australia (52%), and China (13.3–40.7%) (18–20, 25). Qatari parents showed similar findings, namely the younger the child, the greater the vaccine hesitation. For example, parents with children aged 15 had a hesitancy rate of 15.2%, while parents with children aged 12 had a hesitancy rate of up to 21.6% [16]. Among German parents in the KUNO-Kids cohort (ages 1.5 to 5 years old), almost half (49%) expressed parental refusal to vaccinate their children [21]. Thus, parental hesitancy was related to the age of their children. In terms of self-desire, 93.6% of parents in our study were willing to vaccinate for themselves with the current COVID-19 vaccine, which was higher than the 41.8% in a prior study on adults' willingness to take the COVID-19 vaccination during an early Delta variant pandemic in Thailand [31]. The significant increase in the number of infected patients in Thailand during the Omicron variant outbreak may reduce hesitation if parents are aware that vaccination is one of the main strategies of disease prevention. Updating statistics on infected children and children with complications following COVID-19, which demonstrated a higher rate than the preceding epidemic wave, particularly among children aged 5 to 11, may aid in lowering parent concern about immunizing their children.

The main concern among parents and guardians of children under the age of 12 and those over the age of 12 was vaccine side effects, which is similar to many studies such as those carried out in Turkey and Boston, USA, where 40.4–61.5% of participants reported the reason for refusal was avoiding possible vaccine side effects [17], [24]. Moreover, the findings from a systematic review and *meta*-analysis, which revealed that 60.99% (95% CI 48.57–72.30) of parents were unwilling to vaccinate their children for concerns regarding safety and side effects [32]. Half of our participants acknowledged the risk of major side effects of 1 in 1 million, which was similar to a prior study done in the United States, which also found that the chances of a serious adverse reaction were small but significant. Acceptance of the vaccine was lower when the risk of significant side effects was 1/100,000 rather than 1/million (*p*-value < 0.05) [35]. Health care authorities could provide updated statistics on vaccine safety and adverse events after vaccination, which previously revealed that only 0.4 and 0.9 per 1 million of Thai vaccinees experienced anaphylaxis and myocarditis following Pfizer immunization, respectively [6]. This explanation may reduce skepticism among parents who are concerned about their children's safety, as 75.9% of parents in our study were. It may also minimize the 61.5% of parents who would delay their children's COVID-19 immunization for at least one to three months until safety was verified.

Parental vaccination attitudes and perceptions of COVID-19 disease are important factors in vaccinating their children. Our study revealed that parents who believed that the vaccine would be a good way to protect their children from disease and vaccination was important for their child's health were less hesitant to have their children vaccinated. The results were consistent with previous research. Parents who believe that the vaccine can prevent disease are 1.1–14 times more likely to vaccinate their children, according to research conducted in China and six high-income countries, including the United States, Canada, Israel, Japan, Spain, and Switzerland [23], [33]. Furthermore, when parents had been or planned to get vaccinated, they were less likely to refuse vaccination for their child (*p*-value 0.001) [19], [30]. Encourage all hesitant parents to change their attitudes and embrace that the vaccine is safe and effective in preventing COVID-19 infection, hospitalization, and death [34], [35]. Persuade those parents to get the vaccine before giving it to their children.

The source of information is also an important consideration for parents when deciding whether or not to vaccinate their child. According to our studies, information from the government and public health agencies can reduce hesitancy (aOR = 0.53, 95 %CI = 0.29–0.96, *p*-value = 0.035). Moreover, the health care providers and the internet were the top two sources of information for parents to stay up to date on COVID-19 vaccination with similar to a study in Italy [26]. During the pandemic, there was a lot of misinformation and anti-vaccine propaganda. It is proposed that public health officials should collaborate with regard to vaccination campaigns and use social media to alleviate unverifiable rumor and destructive misinformation in order to reduce hesitancy and encourage parents and guardians to make accurate vaccination decisions [32], as information from a trusted doctor could minimize the hesitancy by 53% [25]. Encourage parents to seek information on COVID-19 immunization on a regular basis; this would lessen their concerns by 1.2 times as in a study conducted in Germany [21].

High educational level and socioeconomic status were associated with decreased vaccine hesitancy in our study, particularly among parents with children under the age of 12 years. Educated parents in the United States and Germany had a similar result, showing a greater willingness to vaccinate their children. (aOR 1.99, 95% CI = 1.26–3.34, *p*-value 0.003) [21], [36]. Higher education could provide access to better disease and vaccination information, allowing for better decision-making [26]. Moreover, there was an association between a parent's marital status and parental hesitancy to vaccinate their child. This could be because of Thai culture and how families work. Before making a decision for their child, both parents need to talk about the child's health, their own expectations, and the grandparents expectation, particularly about the COVID-19 vaccination, as it is very new and is currently being debated. If their child has major side effects after getting a shot, it is their responsibility to deal with the consequences. More debate may make things more tense and make parents less likely to let their children get vaccinated, therefore, married parents or guardians were more hesitant than unmarried parents or guardians [37]. Due to low vaccination coverage among children under the age of 12, as well as high hesitation among parents with children under the age of 12, the Thai government should target this population to promote vaccination uptake. Government and health care professionals should develop a strategy that focuses on low education and low socioeconomic parents to vaccinate their children as well as building relationships to spread a positive attitude through trustworthy community coalitions and establishing a successful immunization campaign among children uder the age of 12 [38], [39].

The major limitation of this study is that participants were drawn at random from their parents' social networks via the internet, with convenience sampling from 48 provinces across the country (62%). The majority of participants came from the most populated areas in each region, which included Bangkok and its Metropolitan area, Chiang Mai, Pattaya, Phuket, and Nakorn Ratchasima. Anti-vaxxers and pro-vaxxers may both have participated in this study and have distributed the online questionnaire to the same group of interested people causing bias. The survey was conducted from October to November 2022, during the beginning of the Omicron pandemic in Thailand and the Thailand vaccination program for children over the age of 12. As exposure and results are measured concurrently in cross-sectional study design, a real cause and effect relationship cannot be established. The majority of our respondents (74%) were female. This could be explained by the fact that in most Thai families, the mother is the primary caregiver for their children's health, including vaccinations and other health issues, rather than the father. Future research should collect consecutive data and compare it to earlier results to exhibit a fuller insight into the hesitancy of parents and guardians to vaccinate their children with the first or booster dose. Thus, an effective public health vaccination campaign is likely to require several components, including an education program by health care professionals to raise awareness about the benefits of vaccination on effectiveness, safety, and to increase favorable attitudes about vaccination, which could protect their children's health. Policymakers and public health officials should provide clear information to all hesitant parents and guardians to decrease unwillingness to give their children the current COVID-19 vaccination. This would encourage parents and guardians to overcome vaccine nervousness and embrace a positive attitude towards vaccination and could play a pivotal role in driving a successful Thailand vaccination program for children under the age of 18, achieving herd immunity, and limiting the spread of COVID-19 among Thai children.

## Conclusion

Parents with children under the age of 12 were more hesitant to give the COVID-19 vaccination to their children than parents with children over the age of 12. The majority of parents and guardians were concerned about the safety and adverse effects of vaccines. Parents who believed that the vaccine would protect their children from illness and that vaccination was necessary for their child's health were less hesitant to get their children immunized. Government and public health information might minimize vaccination hesitation among parents and improve vaccine uptake among children. A strategic plan for Thailand vaccination campaign among children should focus on parents with children under the age of 12, parents who refuse to get their COVID-19 immunization, poor education, and low socioeconomic status. A successful Thailand immunization campaign for COVID-19 vaccine could include tailor-made education programs with clear messaging on vaccine safety and efficacy, as well as increasing favorable attitudes among parents and guardians based on up-to-date evidence-based studies and current statistics in Thailand, to reduce vaccination hesitancy among Thai parents. The immunization of school-aged children against COVID-19 is essential, yet vaccine hesitancy among parents and guardians would delay an effective vaccination program.

The authors state that they have no known competing financial interests or personal relationships that could appear to have influenced the work described in this paper: Amornphat Kitro (first author) reports that Chiang Mai University's Faculty of Medicine provided financial and administrative support. Amornphat Kitro (first author) reports an employment affiliation with Chiang Mai University Faculty of Medicine.

## CRediT authorship contribution statement

**Amornphat Kitro:** Conceptualization, Methodology, Formal analysis, Investigation, Writing – original draft, Funding acquisition, Resources. **Wachiranun Sirikul:** Methodology, Formal analysis, Investigation, Funding acquisition. **Ekkamon Dilokkhamaruk:** Writing – original draft. **Gowgeat Sumitmoh:** Writing – original draft. **Sarisa Pasirayut:** Writing – original draft. **Amnart Wongcharoen:** Writing – review & editing. **Jinjuta Panumasvivat:** Writing – original draft, Writing – review & editing. **Krongporn Ongprasert:** Methodology, Funding acquisition, Supervision. **Ratana Sapbamrer:** Methodology, Funding acquisition, Supervision.

## Declaration of Competing Interest

The authors declare that they have no known competing financial interests or personal relationships that could have appeared to influence the work reported in this paper.
