# Supplementary figures and images for: COVID-19 vaccine hesitancy and influential factors among Thai parents and guardians to vaccinate their children
Source: Vaccine X. 2022 Jun 13;11:100182. doi: 10.1016/j.jvacx.2022.100182 (PMC9190183; doi:10.1016/j.jvacx.2022.100182)

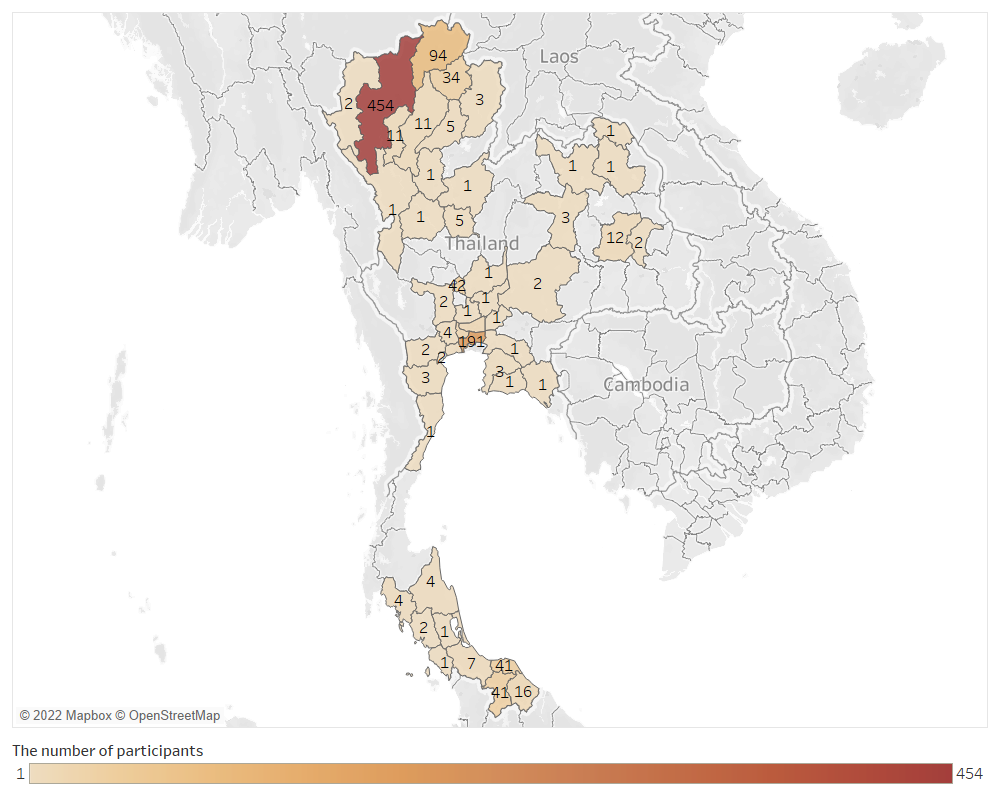

Supplement: Supplementary data 1 [file mmc1.zip › supplementary1.png]
